# Xanthohumol Attenuated Inflammation and ECM Degradation by Mediating HO-1/C/EBPβ Pathway in Osteoarthritis Chondrocytes

**DOI:** 10.3389/fphar.2021.680585

**Published:** 2021-05-04

**Authors:** Ming Zhang, Rui Zhang, Tiansheng Zheng, Zhixi Chen, Guanglin Ji, Fang Peng, Wei Wang

**Affiliations:** ^1^ Department of Orthopedics, Taizhou People’s Hospital, Taizhou, China; ^2^ College of Pharmacy, Gannan Medical University, Ganzhou, China; ^3^ Department of Orthopedics, The First Affiliated Hospital of Gannan Medical University, Ganzhou, China; ^4^ Department of Pathology, The Affiliated Ganzhou Hospital of Nanchang University, Ganzhou, China; ^5^ Department of Hepatology, Taizhou People’s Hospital, Taizhou, China

**Keywords:** xanthohumol, osteoarthritis, ECM degradation, C/EBP, HO-1, mmp-13

## Abstract

Osteoarthritis (OA) is the most frequent and disabling disease in developed countries. The progressive degeneration of articular cartilage characterized as thinner and erosive. Inflammation is well-known to be involved in OA development. However, there are no effective therapeutic strategies to cure it. Xanthohumol (XH) is a natural prenylflavonoid isolated from hops and beer. The protective activity of XH against OA chondrocytes inflammation and ECM degradation is unclear. In this article, we found that XH significantly inhibited inflammatory responses, attenuated catabolic enzymes expression, and ameliorated ECM degradation, as showed by decreased production of NO, PGE2, TNFα, and IL-6, decreased expression of MMP-3/-13 and ADAMTS-4/-5, and increased expression of collagen-II and aggrecan. In addition, XH activated HO-1 signaling and attenuated IL-1β-induced C/EBPβ. XH promoted the interaction between HO-1 and C/EBPβ, inhibiting the nuclear translocation of C/EBPβ. HO-1 knockdown could abrogate the protective effects of XH in IL-1β-treated chondrocytes. Collectively, XH attenuated inflammatory responses and ECM degradation by mediating HO-1 and C/EBPβ signaling pathways in osteoarthritis chondrocytes.

## Introduction

Osteoarthritis (OA), one of the age-related debilitating and degenerative diseases, is often clinically characterized by joint pain, limited movement, and transient morning stiffness (Shamdani et al., 2020). Mechanistically, cartilage degradation, synovial inflammation, and subchondral bone remodeling are involved in the pathological development of OA (Dai et al., 2017). However, the underlying mechanisms are still unclear, and no effective treatments are available to cure OA (Altman and Barthel, 2011).

Inflammation and inflammatory responses have been considered as the main factors contributing to the pathological development of OA. Inflammatory cytokines, such as IL-1β and TNFα, are closely involved in the aberrant metabolism and promote the catabolic activities of cartilage tissues in OA (Nambi, 2020). Expectedly, IL-1β dramatically contributes to the cartilage destruction of OA, which is associated with up regulation of C/EBPβ in chondrocytes (Nishimura et al., 2020). Increasing evidence shows that IL-1β suppresses the production of the component of extracellular matrix (ECM), synthesized by the unique chondrocytes. The possible mechanisms might be associated with the inductive activity of IL-1β on the expression of matrix metalloproteinases (MMPs), thrombin sensitive protein motifs (ADAMTS), cyclooxygenase-2 (COX-2), and inducible nitric oxide synthase (iNOS), which are the main catabolic factors for the destruction of articular cartilage (Wu et al., 2019). Nuclear factor-erythroid 2-related factor (Nrf2)/HO-1 is the critical factor mediating the expression of anti-oxidative enzymes and balancing the redox homeostasis (Zhao et al., 2020). Up regulation of NRF2/HO-1 expression may ameliorate IL-1β-induced inflammation and induction of ECM degradation (Gu et al., 2020). Promisingly, NRF2/HO-1 has become the potential target for the therapeutic management of OA.

Non-steroidal anti-in flammatory drugs (NSAIDs) have been widely used for treating OA, alleviating clinical symptoms, such as pain and swelling. However, they cannot inhibit the progression of OA development but produce many serious adverse effects (Bournia et al., 2017). It is emerging to explore the potential candidates for OA treatment. Recently, morroniside, a bioactive compound isolated from *Cornus officinalis*, has been showed to protect osteoarthritic cartilage by attenuation of inflammation in mice chondrocytes (Park et al., 2021). Engeletin (dihydrokaempferol 3-rhamnoside) is reported to decrease TNFα-induced chondrocytes apoptosis and the production of ROS in rat knee cartilage (Wang et al., 2021). Acacetin can significantly ameliorate IL-1β-induced matrix metalloproteinases expression in chondrocytes by inhibiting NF-κB signaling pathways in mice (Chen et al., 2020a). The therapeutic single compounds for osteoarthritis treatment have been reviewed recently (Lee et al., 2021). Xanthohumol (XH), a natural prenylflavonoid isolated from hops and beer, has been preventing hyaluronan overproduction and subsequent response in the early stage of OA (Stracke et al., 2011). Recently, XH has been reported to reduce the nephrotoxicity induced by cisplatin by activating Nrf2/HO-1 and attenuating NF-κB signaling pathways (Li et al., 2018). However, the biological activity of XH in osteoarthritic chondrocytes is still unclear.

## Materials and Methods

### General

This study was approved by the Institutional Animal Care and Use Committee of Taizhou People’s Hospital and performed in strict accordance with the guideline of Animal Care and Use (No. TZPH2006381962), according to the Declaration of Helsinki Principles.

### Chemicals and Reagents

Xanthohumol (XH) (purity98%) (Cat. no. D108095) was obtained from BIOBW (Beijing, China). Collagenase type II (Cat. no. C2-28) and dimethylsulfoxide (DMSO) (Cat. no. D8418) were purchased from Sigma Aldrich (St Louis, MO, United States). The primary antibodies against C/EBPβ (Cat. no. 3082), HO-1 (Cat. no. 43966), MMP-3 (Cat. no. 14351), GAPDH (Cat. no. 5174S), and the horseradish peroxidase-labelled secondary antibody (Cat. no. 7074S) were purchased from Cell Signaling Technology. MMP-13 (Cat. no. 701287), ADAMTS-4 (Cat. no. PA5-69140), ADAMTS-5 (Cat. no. PA5-32142), collagen-II (Cat. no. MA5-12789), and aggrecan (Cat. no. MA3-16888) were obtained from Invitrogen. Recombinant rat IL-1β (Cat. no. ab9788) purchased from Abcam.

### Rat Knee OA Models

Rat knee OA models were duplicated using a classic osteoarthritic destabilization of the medial meniscus (DMM) model through surgery (Glasson et al., 2007). Simply, eight-week-old male Sprague-Dawley rats (200 g) were anesthetized with 3% (w/v) pentobarbital (30 mg/kg) intraperitoneally. Then, surgery was conducted to cut the medial meniscus tibial ligament through the medial patellar tendon of the capsule in the right knee joints. The sham group was performed without cutting off the medial meniscus tibial ligament in the left knee joints. After eight weeks of post-operation, rats were sacrificed, and joint cartilages were collected for gross observation, histochemical examination, and immunohistochemical evaluation. The low and high doses of XH for treating rats OA were 5.64 mg/kg and 16.9 mg/kg, respectively, which were reported to be corresponding to the values of 60 mg and 180 mg XH, respectively, for humans with a body weight of 66 kg (Legette et al., 2012). Different doses of XH were dissolved in 0.5% DMSO. The sham group received the same volumes of dosing vehicle as the treating groups.

### Cell Culture

Under sterile conditions, the articular cartilage of the articulatio genus, terminal femur, and upper tibia was collected, cut into small pieces, and digested with 0.25% pancreatic enzyme and 0.2% collagenase II at 37°C for 4 h. Cells were cultured in Dulbecco’s modified Eagle’s minimum essential medium (DMEM) (low glucose) (Life Technologies, NY, United States) supplemented with 10% fetal bovine serum (FBS), penicillin, and streptomycin (Life Technologies) at 37°C with 5% CO_2_. 24 h later, the fresh complete culture medium was used for replacement. The cells employed for all the experiments were the second or the third generation of the primary cartilage cells, ensuring consistent cell phenotypes. XH dissolved in DMSO was prepared, and the final concentration of DMSO was 0.1% in the culture medium.

### Cell Viability Assays

The CCK8 kit (Cat. no. C0038) (Beyotime, Shanghai, China) was used for testing the chondrocytes viability according to the manufacturer’s instructions. Chondrocytes were grown in 96-well plates at a density of 5000 cells/well for 24 h. Different concentrations of XH (0, 5, 10, 20, 40, and 80 μM) were used to pretreat the chondrocytes for 24 and 48 h, respectively. Then, CCK-8 solution (10 μl) was added into each well and incubated at 37°C for 4 h. After that, the absorbance was detected at the wavelength of 450 nm by using a microplate system (Leica microsystems, Germany).

### Detection of NO, PGE2, TNFα, and IL-6

Chondrocytes were grown in 6-well plates, and XH was added to treat for 24 h. Then, cells were treated with the recombinant IL-1β for another 24 h. The levels of NO metabolite nitrite were detected as the production of NO by using sodium nitrite as the standard (Au et al., 2007). Simply, 100 μl of the culture supernatant was permitted to react with Griess reagent in an equal volume in 96-well microplate at room temperature for 10 min in the dark. The absorbance was detected at the wavelength of 540 nm by using a microplate system (Leica microsystems, Germany). The productions of PGE2, TNFα, and IL-6 were determined by employment of ELISA kits (Beyotime, Shanghai, China) according to the manufacturer’s instructions.

### Cell Transfection

HO-1 siRNA and scramble siRNA (negative control), obtained from RiboBio (Guangzhou, China), were used for investigating the roles of HO-1 knockdown in the inflammatory responses in chondrocytes. Cells were cultured in 6-well plates at a density of 1×10^5^ cells/well. Once they reached 60% confluence, cells were used for the subsequent transfections. According to the manufacturer’s instructions, transfections of HO-1 siRNA and negative control were performed by using lipofectamine 3000 reagent (Thermo Fisher Scientific, Inc.) for 36 h at 50 nM. Western blot assays were used for determining the transfection efficiency.

### Western Blotting

RIPA lysis buffer (Beyotime Institute of Biotechnology) containing 1% PMSF was used to lyse cells. BCA protein assay kit (cat. no. 23250) (Pierce Biotechnology) was employed to quantify the total protein. 25 μg protein/lane was separated *via* gel electrophoresis and transferred to polyvinylidene fluoride membrane (Bio-Rad, CA, United States). It was blocked by tris-buffered saline supplemented with 5% nonfat milk for 1 h at room temperature. After washing three times with TBST (Tris-buffered saline containing Tween 20), the membrane was cut and incubated with the corresponding primary antibody at 4°C overnight. Then, it was washed with TBST for three times and incubated with horseradish peroxidase-labelled secondary antibody for 1 h at room temperature. Protein bands were detected using the enhanced chemiluminescence detection system (Bio-Rad Laboratories) and Quantity One software v4.6.2 (Bio-Rad Laboratories).

### Co-immunoprecipitation Assay

Chondrocytes were harvested by the immunoprecipitation lysis buffer. Then, they were centrifugated at 12,000 rpm for 30 min 10% of chondrocytes lysates were kept and used as the input. The remained proteins were immunoprecipitated by incubating with normal goat IgG or HO-1 in immunoprecipitation washing buffer at 4°C overnight. Next, they were incubated with the pre-cleared protein A-sepharose beads at 4°C for 2 h. The eluted samples were then subjected to Western blot as mentioned above.

### Immunofluorescence

Chondrocytes were grown on glass coverslips for 24 h. After rinsing with PBS for three times, cells were fixed with 4% paraformaldehyde for 15 min at room temperature and rinsed with PBS again. 0.1% Triton X-100 was used to infiltrate cell and nuclear membranes for 5 min at room temperature. Then, cells were blocked by 5% protease-free bovine serum albumin (BSA) for 1 h at room temperature, rinsed with PBS, and incubated with the corresponding primary antibody at 4°C overnight. Cells were washed with PBS and incubated with fluorescein-conjugated goat anti-rabbit IgG antibody for 1 h at room temperature. Subsequently, cells were mounted in medium containing DAPI (Invitrogen) after washing with PBS. A confocal laser scanning microscope (Leica Microsystems) was employed to observe the slides, and the fluorescence intensity was detected by using ImageJ software 2.1 (Bethesda, MD, United States).

### Statistical Analysis

All experiments were performed in triplicate and data are presented as the mean ± standard error of the mean. SPSS 20.0 software (IBM Corp., Armonk, NY, United States) was used for statistical analysis. One-way ANOVA and followed by Tukey’s post hoc test was used to make statistical comparisons between multiple groups. An unpaired Student’s *t*-test was used to make statistical comparisons between two groups. *p* < 0.05 was considered to indicate a statistically significant difference.

## Results

### XH Exhibited Protective Activity Against OA Development in Rats

**FIGURE 1:**
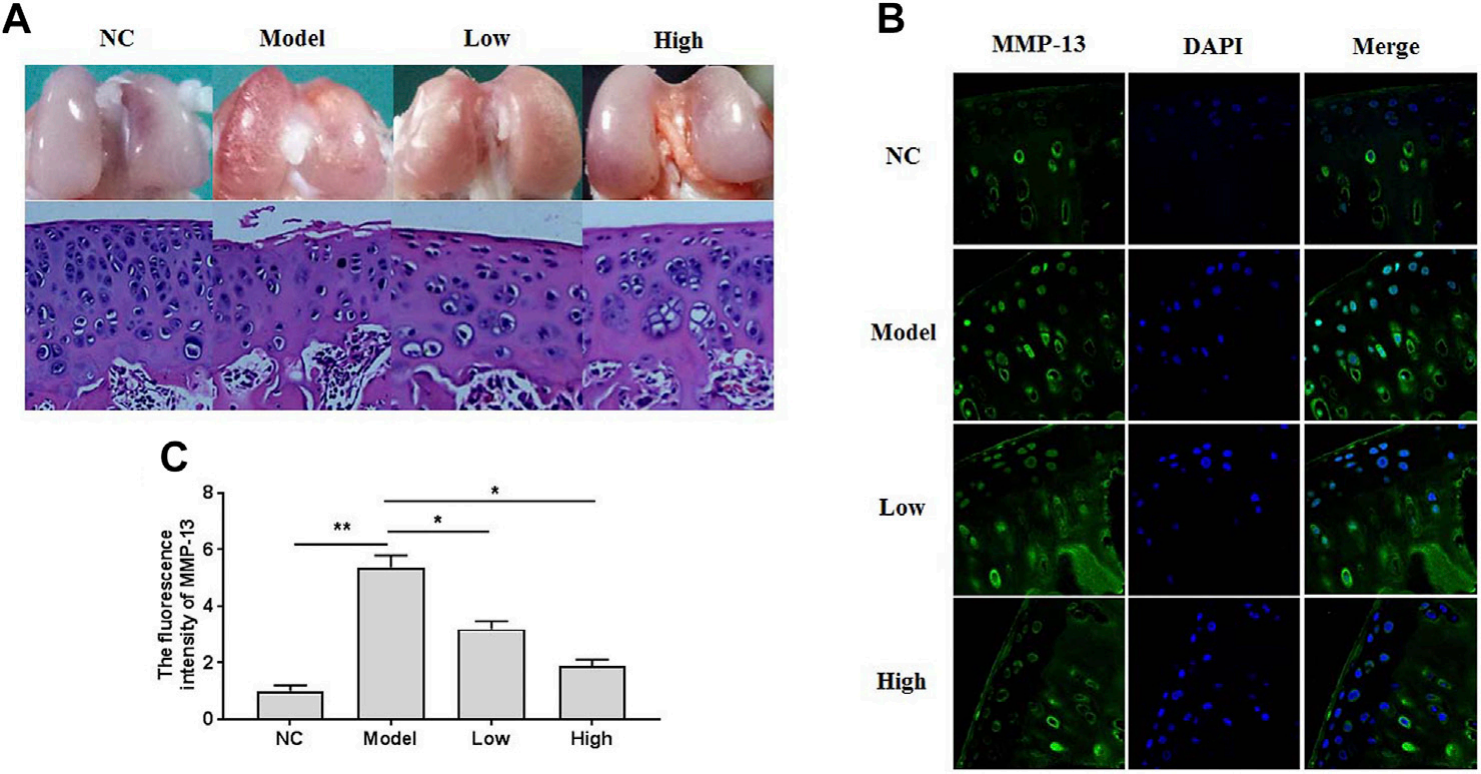
The gross observation, histomorphological examination, and immunofluorescence assays in the knee articular cartilage. **(A)** The gross observation and histomorphological examination (HE staining) of articular cartilage. **(B)** The immunofluorescence study of MMP-13 in cartilage. **(C)** The summary data of fluorescence intensity of MMP-13 *in situ*. All experiments were performed in triplicate and data are presented as the mean ± standard deviation. ^*^ *p* < 0.05 and **p < 0.01. NC, negative control; Model, the model group; Low, the group treated with XH (5.64 mg/kg); High, the group treated with XH (16.9 mg/kg).

To investigate the effects of XH on OA development, a surgical rat DMM model was duplicated and treated with XH (5.64 mg/kg and 16.9 mg/kg) for 8 weeks after operation by oral administration. As showed in Figure 1A, gross observation and histomorphological examination were studied. No obvious damages were seen in the negative control group. In contrast, the rough surface of cartilage with some erosion was observed in the model group. Consistently, the decreased number and disorder distribution of chondrocytes and decreased thickness of articular cartilage were found in the model group by hematoxylin-eosin (HE) staining. XH could protect articular cartilage from damage, as indicated by better cartilage surface, increased cell number, and thicker cartilage dose dependently. The immunofluorescence study of MMP-13 *in situ* (Figure 1B) showed that oral administration of XH might significantly decrease the fluorescence intensity (Figure 1C) in a dose-dependent manner, compared with that in the model group. These indicated that XH exhibited protective effects against de-structure in articular cartilage, which might be possibly associated with down regulation of MMP-13 expression in chondrocytes.

### Effects of XH on Cell Viability

**FIGURE 2:**
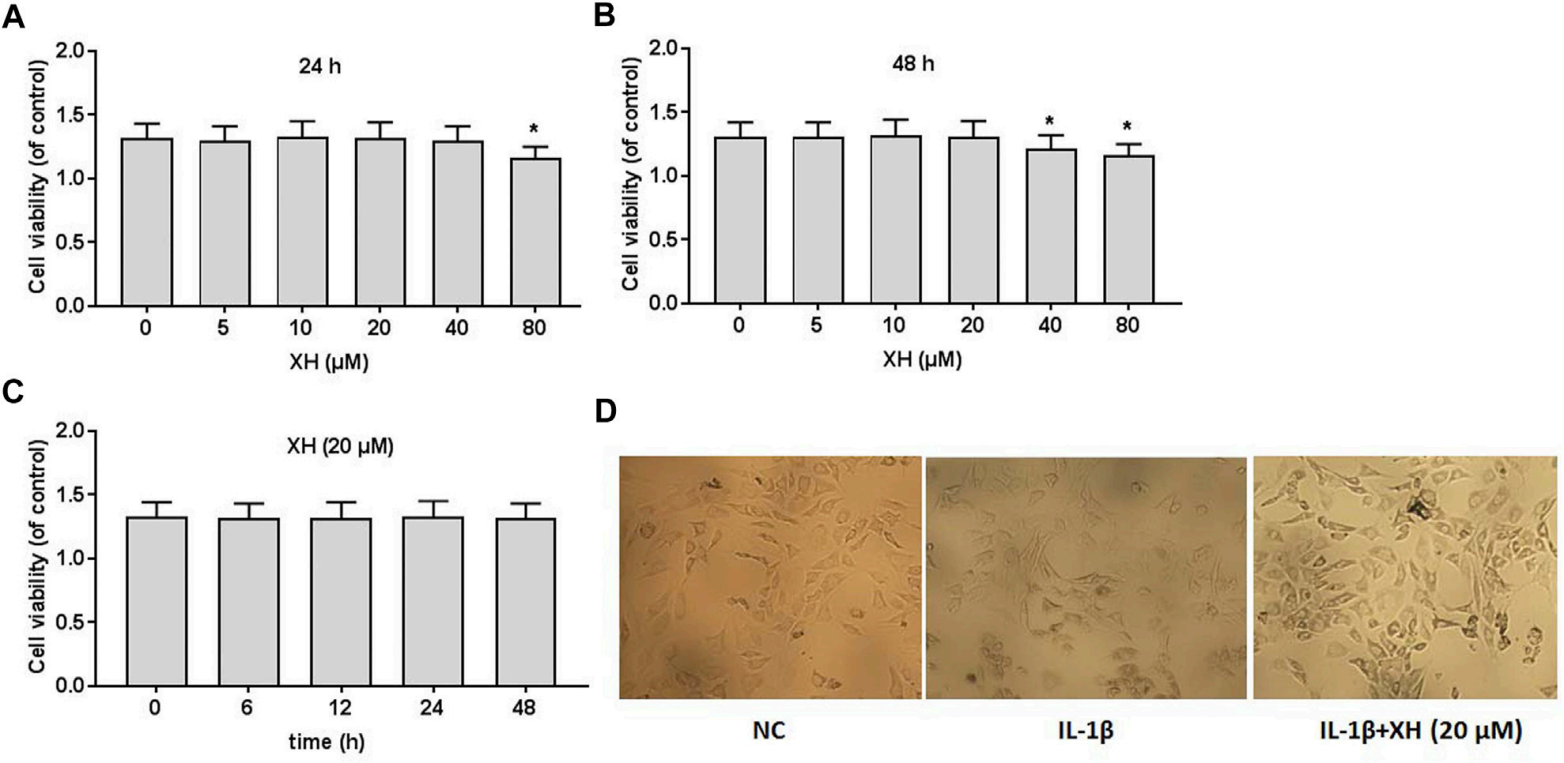
Cell viability of XH on rat chondrocytes. CCK8 assays were conducted to determine the effects of XH (0, 5, 10, 20, 40, and 80 μM) in 24 h **(A)** and 48 h **(B)**, respectively. **(C)** The effects of XH (20 μM) on cell viability in 48 h. **(D)** The cell morphology was observed after administration with IL-1β (10 ng/ml) with or without XH (20 μM) in 48 h. All experiments were performed in triplicate and data are presented as the mean ± standard deviation. ^*^ *p* < 0.05 and **p < 0.01.

To detect the effects of XH on the viability of rat chondrocytes, the different doses XH (0, 5, 10, 20, 40, and 80 μM) were administered to the cultured cells for 24 and 48 h, respectively, before cell viability was detected using CCK8 assays. As indicated in Figures 2A,B XH did not produce any cytotoxicity when the concentration of XH was less than 20 μM. After 48 h incubation, XH at the dose of 40 μM slightly reduced the viability of chondrocytes, compared with that after 24 h incubation. At the dose of 20 μM, XH did not exhibit toxic effects on chondrocytes in 48 h (Figures 2C,D).

### XH Decreased the Productions of Inflammatory Cytokines in IL-1β-Treated Chondrocytes

**FIGURE 3:**
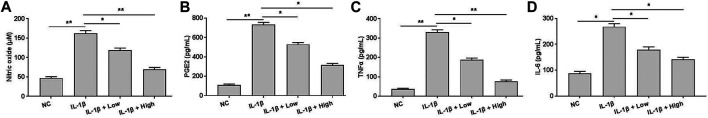
Effects of XH on the production of NO, PGE_2_, TNFα, and IL-6 in IL-1β-treated chondrocytes. **(A)** The production of NO was determined. **(B)** The production of PGE_2_ was determined. **(C)** The production of TNFα was determined. **(D)** The production of IL-6 was determined. All experiments were performed in triplicate and data are presented as the mean ± standard deviation. *p < 0.05 and **p < 0.01. NC, negative control; Low, XH (5 μM); High, XH (20 μM).

To investigate the effects of XH on chondrocytes regarding inflammation, cells were pretreated with IL-1β (10 ng/ml) and XH (5 and 20 μM) for 24 h. The productions of inflammatory cytokines, including NO, PGE_2_, TNFα, and IL-6 were determined. As shown in Figure 3, IL-1β could significantly stimulate the expression of NO (Figure 3A), PGE_2_ (Figure 3B), TNFα (Figure 3C), and IL-6 (Figure 3D). In contrast, XH exhibited protective activity and compromised the effects of IL-1β, decreasing the productions of these inflammatory cytokines in chondrocytes.

### XH Ameliorated IL-1β-Induced ECM Degradation in Chondrocytes

**FIGURE 4:**
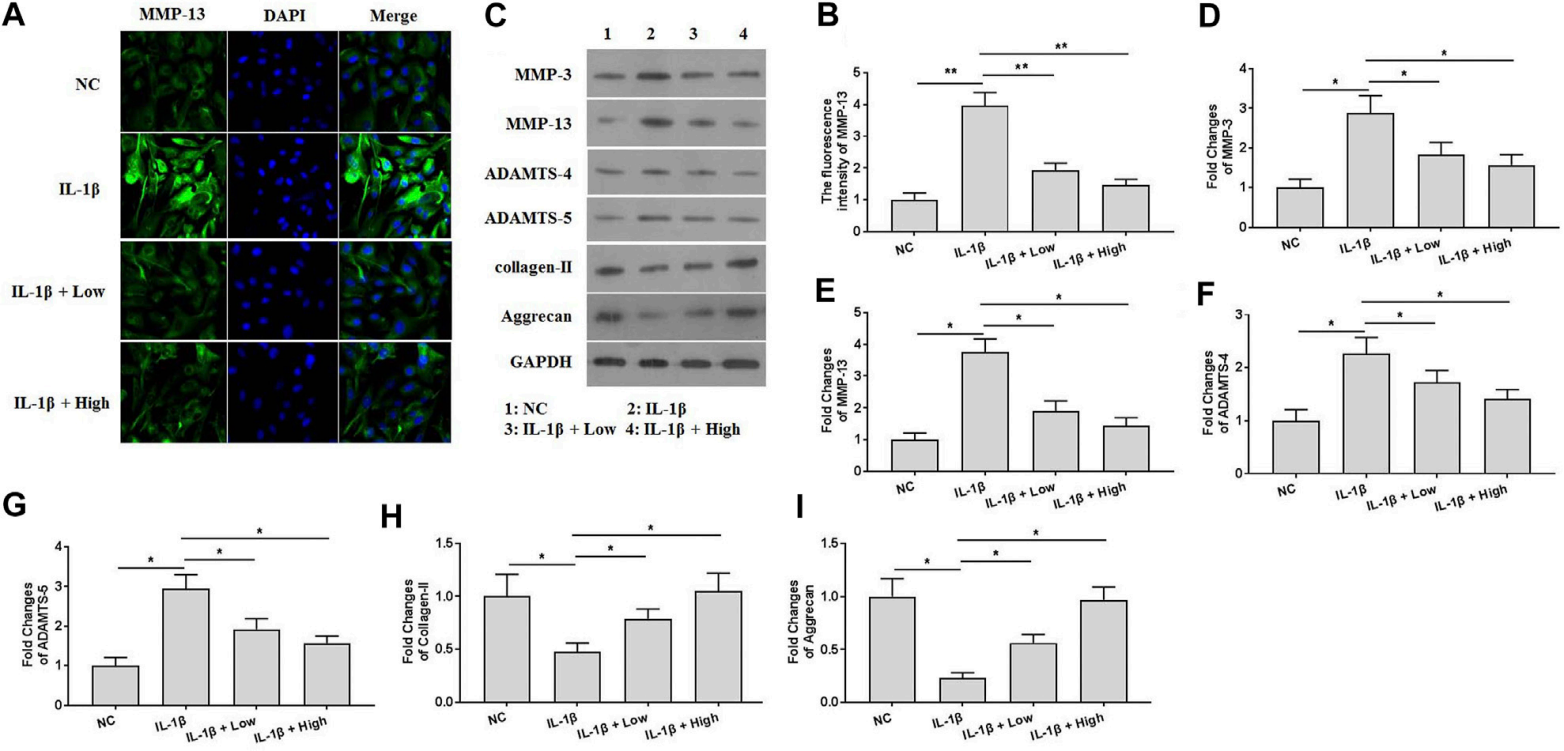
XH attenuated ECM degradation in IL-1β-treated chondrocytes. **(A)** The immunofluorescence study of MMP-13 in IL-1β-treated chondrocytes. **(B)** The summary data of fluorescence intensity of MMP-13. **(C)** The proteins expression of MMP-3, MMP-13, ADAMTS-4, ADAMTS-5, collagen-II, and aggrecan were detected by western blotting. **(D–I)** were the quantified values of tested proteins. All experiments were performed in triplicate and data are presented as the mean ± standard deviation. *p < 0.05 and **p < 0.01. NC, negative control; Low, XH (5 μM); High, XH (20 μM).

To explore the protective effects of XH against ECM degradation *in vitro*, immunofluorescence and western blotting assays were performed. The results of immunofluorescence assays (Figures 4A,B) showed that XH could effectively attenuate the expression of MMP-13, which was significantly induced by IL-1β. Consistently, IL-1β also increased the expression of the proteins of MMP-3, MMP-13, ADAMTS-4, and ADAMTS-5 and decreased the expression of collagen-II and aggrecan in chondrocytes (Figures 4C,D). Administration of XH could ameliorate IL-1β-induced up regulation of MMP-3, MMP-13, ADAMTS-4, and ADAMTS-5 expression and down regulation of collagen-II and aggrecan expression in chondrocytes.

### XH Inhibited the Activity of C/Ebpβ by Stimulating HO-1 Expression in IL-1β-Treated Chondrocytes

**FIGURE 5:**
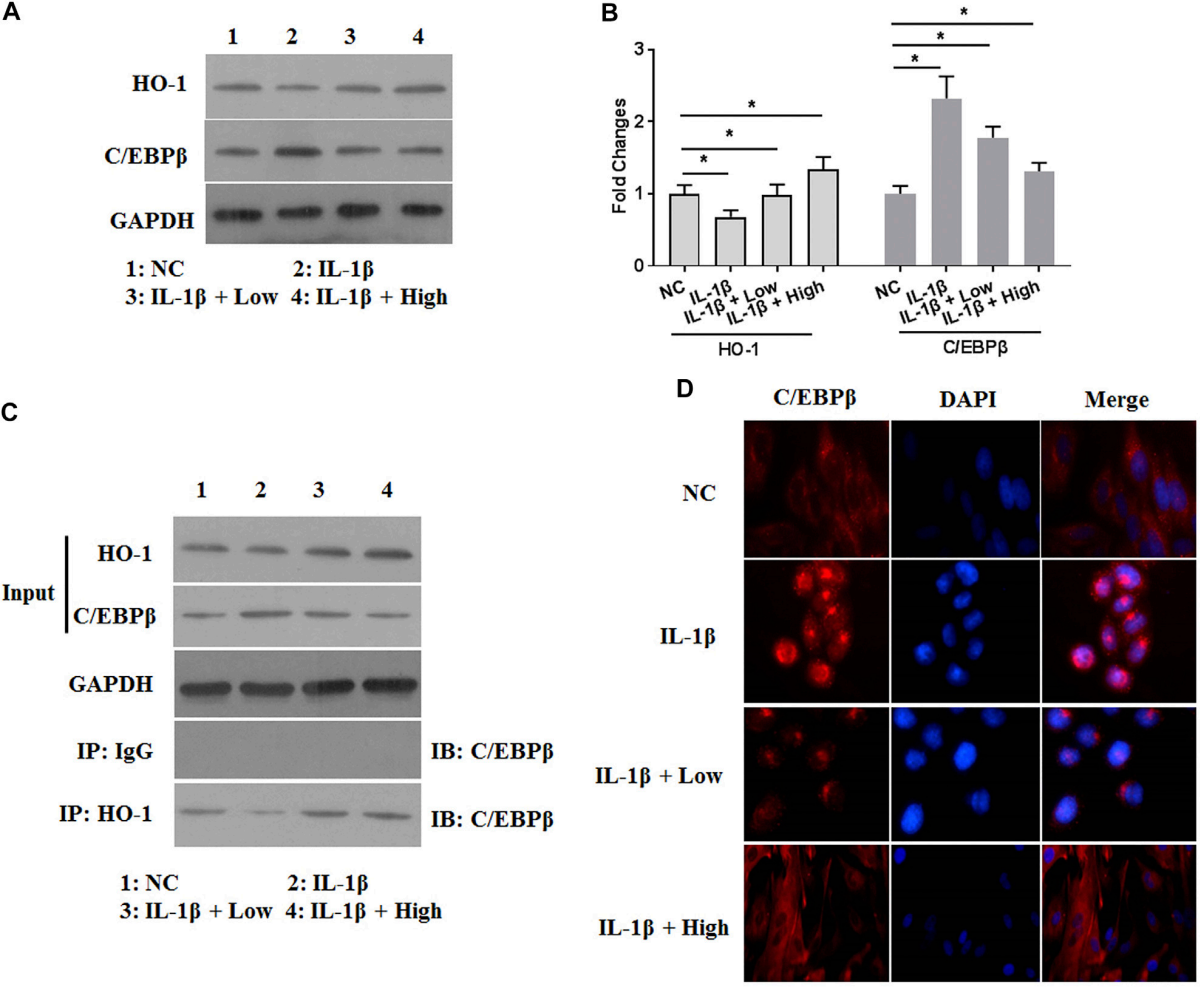
XH increased the expression of HO-1 and attenuated the expression of C/EBPβ in IL-1β-treated chondrocytes. **(A)** The proteins expression of HO-1 and C/EBPβ were detected by western blotting. **(B)** The quantified values of tested proteins were indicated. **(C)** The co-immunoprecipitation of HO-1 and C/EBPβ was studied. **(D)** The immunofluorescence study of C/EBPβ in IL-1β-treated chondrocytes. All experiments were performed in triplicate and data are presented as the mean ± standard deviation. *p < 0.05 and **p < 0.01. NC, negative control; Low, XH (5 μM); High, XH (20 μM).

To further investigate the catabolic responses of ECM in IL-1β-treated chondrocytes, the effects of XH on the activity of IL-1β-activated C/EBPβ were involved. Interestingly, the expression of C/EBPβ was increased by IL-1β (Figures 5A,B). XH could effectively attenuate IL-1β-activated C/EBPβ expression in chondrocytes. XH has been reported to be an activator for NRF2/HO-1 signaling (Zimmermann et al., 2015). Whether HO-1 expression was involved in the protective activity of XH against IL-1β-induced C/EBPβ in chondrocytes, western blotting assays were conducted. The protein expression of HO-1 was detected (Figures 5A,B). IL-1β decreased HO-1 expression. XH reversed it effectively. Co-immunoprecipitation assays were conducted. XH promoted the interaction between HO-1 and C/EBPβ (Figure 5C). The results of immunofluorescence assays (Figure 5D) demonstrated that IL-1β promoted translocation of C/EBPβ into the nucleus, and XH could block C/EBPβ translocation induced by IL-1β in chondrocytes. These suggested that XH inhibited the activity of C/EBPβ by stimulating HO-1 expression in IL-1β-treated chondrocytes.

### XH Ameliorated IL-1β-Induced ECM Degradation by Stimulating HO-1 Activity in Chondrocytes

**FIGURE 6:**
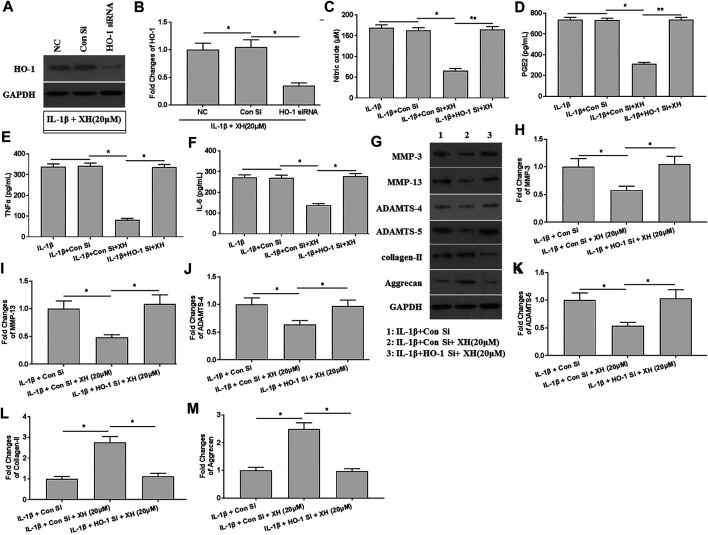
XH attenuated ECM degradation induced by IL-1β via up regulation of HO-1 in chondrocytes. **(A)** The expression of HO-1 was detected in HO-1 siRNA-transfected chondrocytes. **(B)** The quantified values of tested protein were indicated. The productions of inflammatory cytokines NO **(C)**, PGE_2_ **(D)**, TNFα **(E)**, and IL-6 **(F)** in HO-1 siRNA-transfected chondrocytes were determined by ELISA. **(G)** The proteins expression of MMP-3, MMP-13, ADAMTS-4, ADAMTS-5, collagen-II, and aggrecan were detected in HO-1 siRNA-transfected chondrocytes. **(H–M)** were the quantified values of tested proteins. All experiments were performed in triplicate and data are presented as the mean ± standard deviation. ^*^ *p* < 0.05 and **p < 0.01. NC, negative control; Con Si, control siRNA.

To investigate the roles of XH-mediated HO-1 expression in IL-1β-induced ECM degradation, HO-1 was knockdown by siRNA. The decreased expression of HO-1 was observed, indicating successful transfection (Figures 6A,B). In HO-1-knockdown chondrocytes, the productions of NO, PGE_2_, TNFα, and IL-6 induced by IL-1β were not significantly down regulated by XH (Figures 6C–F). Similarly, the expression of the proteins of MMP-3, MMP-13, ADAMTS-4, ADAMTS-5, collagen-II, and aggrecan in IL-1β-treated chondrocytes showed no difference those in the negative control group (Figures 6G–M). These suggested that the protective mechanisms of XH against ECM degradation in cartilage might be associated with attenuation of inflammation and C/EBPβ expression induced by IL-1β.

## Discussion

Maintenance of homeostasis in articular cartilage is tightly mediated by the anabolic and catabolic players in the metabolism of cartilage matrix. Recently, several inflammatory mediators have been demonstrated to be the major players in joint articular matrix degradation by up regulating the expression of MMPs (Nguyen et al., 2017), which are the important proteolytic enzymes degrading ECM and contributing the turnover of cartilage. Three main therapeutic agents including NSAIDs, disease-modifying OA drugs, and steroid and biological response modifiers have been developed for the main treatment strategy of OA (Saeki et al., 2012). However, long-term administration of these agents may produce serious side effects. Natural sources have been interested scientifically for developing novel managing strategy (Jayakumar et al., 2020). In this study, we found that XH could effectively decrease the productions of inflammatory cytokines NO, PGE_2_, TNFα, and IL-6, stimulate HO-1 expression, attenuate the activity of C/EBPβ, promote the interaction of HO-1 and C/EBPβ, and inhibit C/EBPβ nuclear translocation, resulting in amelioration of catabolic enzymes expression in IL-1β-treated chondrocytes.

Chronic, low-grade, and unregulated inflammation has been involved in the pathophysiological development of several human diseases, including osteoarthritis. Many studies showed that high levels of inflammatory responses are associated with OA development in human or experimental animal models and administration of anti-inflammatory agents is clinically implicated in OA treatment (Ansari et al., 2020). IL-1β and TNFα are often used to be stimulators to primary chondrocytes or cartilage explants, mimicking pathological conditions *in vivo* (Daheshia and Yao, 2008). Often, they up regulate the expression of many catabolic factors, such as COX-2, PGE_2_, IL-6, iNOS, MMPs, and ADAMTSs, and down regulate the expression of collagen-II and aggrecan (Ansari and Haqqi, 2016). In our study, we employed IL-1β as the stimulator to the primary isolated chondrocytes to duplicate cell models. As expected, IL-1β up regulated the expression of NO, PGE_2_, IL-6, MMP3, MMP-13, and ADAMTS-4/5 in chondrocytes and down regulated the expression of collagen-II and aggrecan.

MMPs are the collagenases to degrade collagen in the cartilage and bone. Aberrant expression of MMPs is linked to OA progression, and MMPs have become the potential targets for developing specific inhibitors implicated in clinic (Li et al., 2017). MMP-13 is the major enzyme to induce the degradation of collagen, particularly collagen (Knäuper et al., 1997). MMP-13 is the factor responsible for the early onset of OA, and its increased expression remained elevated throughout the 10 weeks study (Pickarski et al., 2011). Interestingly, MMP-13 acts as a biomarker to initiate the degradation of various downstream matrix and collagen components, and it has been comprehensively reviewed (Li et al., 2017). ADAMTS-5 is the major aggrecanase, a critical factor associating with OA pathogenesis, and it is much more active than ADAMTS-4 catalytically (Fushimi et al., 2008). In our study, we found that the expression of MMP-13 was significantly elevated *in vivo* and *in vitro* by immunofluorescence and western blotting assays. The proteins expression of ADAMTS-4 and ADAMTS-5 were also increased in IL-1β-treated chondrocytes.

Mechanistically, the regulation of the inflammatory cytokines, COX-2, iNOS, MMPs, and ADAMTSs is tightly mediated by inflammatory signaling pathways (Sun et al., 2020). Treatment with IL-1β may trigger a cascade of events and induce the activation of C/EBPβ expression in chondrocyte (Tsushima et al., 2012). It has been reported that C/EBPβ in the nucleus can bind to the promoter region of MMP-13 and induce its expression (Hayashida et al., 2009). Thus, IL-1β up regulated MMP-13 expression by promoting the recruitment of C/EBPβ to the promoter of MMP-13. In our study, we consistently demonstrated that IL-1β activated C/EBPβ expression and promoted C/EBPβ nuclear translocation in rat chondrocytes.

Natural products have gained increasing attention due to novel structures for drug development. *β*-Hydroxyisoamylshikonin, a natural naphthoquinone, has been reported to exhibit anti-inflammatory and anti-oxidative activity. *β*-Hydroxyisoamylshikonin can decrease the expression of NO, PEG2, IL-6, TNFα, iNOS, ADAMTS-5, and MMP13 in chondrocytes by down regulation of NF-κB pathway and up regulation of NRF2/HO-1 pathway (Chen et al., 2020b). Hyperoside also decrease the productions of inflammatory cytokines, down regulates the expression of MMPs and ADAMTS-5, and mediates the activity of NF-κB/MAPK and NRF2/HO-1 signaling pathways (Sun et al., 2021). XH is a prenylated chalcone isolated from the hop plant and shows anti-inflammatory, anti-oxidative, anti-proliferative, and anti-angiogenic activity (Harikumar et al., 2009; Krajka-Kuźniak et al., 2020). Consistently, our study showed that XH could decrease the productions of the inflammation cytokines. Interestingly, XH has also been an activator of NRF2/HO-1 signaling (Bai et al., 2019; Krajka-Kuźniak et al., 2020). XH exhibits free radicals scavenging activity by activating NRF2/HO-1 signaling, preventing neuro-degeneration (Wang et al., 2019). Our study found that XH increased the expression of HO-1 in chondrocytes.

It has been reported that the expression of NRF2/HO-1 is showed to be negatively correlated with C/EBPβ in neonatal satellite cell (Zecchini et al., 2019). In HO-1 knockout mice, the expression of C/EBPβ is significantly increased in myelocytes (Bukowska-Strakova et al., 2017). These indicate that C/EBPβ might be a downstream factor of HO-1. Our study revealed that XH promoted the binding of HO-1 to C/EBPβ and inhibited C/EBPβ nuclear translocation. To further investigate the roles of XH in protection against IL-1β-induced inflammatory responses and ECM degradation, HO-1 siRNA was employed to knockdown the expression of HO-1. Our study showed that HO-1 knockdown could abrogate the protective effects of XH on IL-1β-treated chondrocytes.

However, there are some limitations in this study. The selected doses were not obtained by our previous study but by the reference from Legette et al. (2012), who calculated them according to the FDA-approved Guidance for Industry. It is necessary to re-confirm them under the different environment. Recently, it has been demonstrated that DMSO exhibits anti-inflammatory and anti-oxidative activities (Kim et al., 2006; Cheleschi et al., 2018). In our study, DMSO was used as the vehicle to dissolve XH. The negative control group in rat OA models was applied to administration with the same volume vehicle. *In vitro*, the effects of 0.1% DMSO on the productions of inflammatory cytokines were investigated, and they showed no significant changes (Supplementary Figure S1). This might be associated with its low concentration. Then, the remaining experiments were conducted without consideration of DMSO influence. However, DMSO still exhibited potential activity on inflammatory and oxidative stress. More efforts are still needed for further confirmation.

## Conclusion

Collectively, XH ameliorated IL-1β-induced inflammatory responses and ECM degradation by activation of HO-1 expression and inhibition of C/EBPβ activity in chondrocytes.

## Data Availability

The original contributions presented in the study are included in the article/Supplementary Material, further inquiries can be directed to the corresponding author.
